# An Approach for Predicting Essential Genes Using Multiple Homology Mapping and Machine Learning Algorithms

**DOI:** 10.1155/2016/7639397

**Published:** 2016-08-30

**Authors:** Hong-Li Hua, Fa-Zhan Zhang, Abraham Alemayehu Labena, Chuan Dong, Yan-Ting Jin, Feng-Biao Guo

**Affiliations:** ^1^Center of Bioinformatics, School of Life Science and Technology, Key Laboratory for Neuroinformation of the Ministry of Education, University of Electronic Science and Technology of China, Chengdu, China; ^2^Center of Information in Biomedicine, University of Electronic Science and Technology of China, Chengdu, China

## Abstract

Investigation of essential genes is significant to comprehend the minimal gene sets of cell and discover potential drug targets. In this study, a novel approach based on multiple homology mapping and machine learning method was introduced to predict essential genes. We focused on 25 bacteria which have characterized essential genes. The predictions yielded the highest area under receiver operating characteristic (ROC) curve (AUC) of 0.9716 through tenfold cross-validation test. Proper features were utilized to construct models to make predictions in distantly related bacteria. The accuracy of predictions was evaluated via the consistency of predictions and known essential genes of target species. The highest AUC of 0.9552 and average AUC of 0.8314 were achieved when making predictions across organisms. An independent dataset from* Synechococcus elongatus*, which was released recently, was obtained for further assessment of the performance of our model. The AUC score of predictions is 0.7855, which is higher than other methods. This research presents that features obtained by homology mapping uniquely can achieve quite great or even better results than those integrated features. Meanwhile, the work indicates that machine learning-based method can assign more efficient weight coefficients than using empirical formula based on biological knowledge.

## 1. Introduction

Essential genes are those genes which are indispensable for the basic activities of organisms under certain growth conditions [1]. The proteins coded by essential genes are considered to carry out the fundamental biological functions. Therefore, these essential genes are regarded as the basis of life [2]. Knowing more about necessity of genes can help researchers find out the existence form of microbes [3], construct the minimal gene subset [4], discover potential drug targets, and design reasonable and effective drugs to resist microbial pathogens [5]. In addition, these genes are more inclined to be related to basic cellular processes such as duplication and translation, which would lead essential genes to be more stringent than nonessential genes when negative (purifying) selection occurs [6, 7]. Because of their tremendous functions in cells, the research of essential genes has become hotspot in bioinformatics and genomics. A series of experimental approaches such as single gene knockouts [8], conditional knockouts [9], RNA interference [10], and transposon mutagenesis [11] have been provided to identify microbic essential genes. While experimental techniques may be reliable, these methods have significant shortcomings, such as high cost and long duration.

As an alternative way, computational methods do not have the above-mentioned drawbacks. Hence, some researchers attempt to use computational techniques combining with biological characteristics to identify essential genes. To some extent, some of these methods have obtained satisfactory results. Deng et al. trained classifier on the basis of several biological features including intrinsic and context-dependent genomic features. The results of their method yielded AUC scores between 0.86 and 0.93 through tenfold cross-validation test in the same organism and 0.69 to 0.89 for cross organism predictions [12]. Song et al. used *Z*-curve and some other features which derived from sequences combining with their linear method to predict essential genes. They acquired AUC scores between 0.8042 and 0.9319 in 12 organisms [13]. These biological features often used can be summarized as three types: intrinsic genomic features like GC content, derived features from sequence like codon adaption index, experimental data like gene expression profile, as well as other features like gene ontology and functional gene network [14–18]. Although these features are associated with gene essentiality, the majority of them cannot be collected or available in most of microbes, nor does every feature have high predictive power in identification of essential genes. Besides, these features may increase biological redundancy. Therefore, most of the methods based on various biological characteristics just could be developed in merely a handful of species. However, essential genes tend to be conserved during the long-term evolution [19]; thus sequence alignment is of great significance for molecular function prediction [20]. Considering these factors, our group previously developed a universal tool named Geptop (gene essentiality prediction tool based on orthology and phylogeny) to predict gene essentiality [21]. This approach uses reciprocal best hit (RBH) method to obtain the results of homology mapping and considers the distance of phylogeny as the weight of orthology variables. Through a series of tests, Geptop, a method designed only based on biological knowledge, has obtained quite better results than those based on integrated features.

In this study, we attempted to investigate the optimized weight and find whether it can further improve the predictions or not. In recent years, the machine learning-based methods have shown significant performance in many prediction researches [22]. Therefore, we put forward a new approach to identify essential genes based on multiple homology mapping and machine learning technique. For a given organism, the greatest weight is the evolutionary distance between it and its closest related organism in Geptop. However, in our method, it was measured by the feature with the best ability to distinguish positive and negative sample sets.

## 2. Materials and Methods

### 2.1. Data Sources

Annotations of essential genes were downloaded from the latest version of Database of Essential Genes (DEG) at http://tubic.tju.edu.cn/deg/. 39 bacterial essential gene sets are included in DEG database, but not all of them are reliable because of the limitations of wet-lab technologies. Additionally, an organism may have different batches of data accompanying with different accuracy, and some genomes contain many conditional essential genes which are specific in these organisms. Therefore, we excluded those inappropriate datasets and finally chose 25 essential gene sets as positive datasets. Meanwhile, we downloaded another annotation data of* Escherichia coli* K_12* (E. coli)* from Profiling of* E. coli* Chromosome (PEC) [23] for extra study. Those genes, which cannot be found in essential gene sets, are regarded as nonessential genes and are used to construct negative datasets for each organism. Therefore, each gene of the target species was assigned a Boolean value to label the essentiality (essential: +1; nonessential: −1). The complete protein coding sequences with fasta format of 25 organisms were obtained from NCBI Genomes. We listed all species used in this work in Table 1. In order to describe these species conveniently, we gave an abbreviated name for each of them (Table 1).

### 2.2. Homology Mapping

Essential genes tend to be more conserved than nonessential genes in the process of long-term evolutionary. Hence, they should be kept in most of the bacteria [24]. This property of essential genes constitutes the basis of our method. In our work, we used the method of reciprocal best hit (RBH) to identify orthologs between the selected organisms through pair-wise comparison. For two given organisms, one was used as the query species Q and the other was used as the referential species R. Firstly, we queried an CDS_*i*_ (coding DNA sequence) of Q against all CDS_*s*_ in R by Blastp program with a default *E*-value threshold of 10 and yielded a set of hits {M}. Then, we queried CDS_*j*_ with the lowest *E*-value in {M} against all CDS_*s*_ in Q by the same way and yielded a set of hits {N}. A pair of proteins (CDS_*i*_, CDS_*j*_) are considered orthologs if we queried CDS_*i*_ with the lowest *E*-value in {N}. Therefore, CDS_*j*_ is assumed as orthologous-essential gene if the referential gene is annotated as essential by experimental methods in species R. And the value of sample CDS_*i*_ for feature R is marked as 1 if CDS_*i*_ has orthologous-essential gene in species R. Otherwise, it is marked as 0. Finally, a gene can be represented by a series of binary values. Specially, for training sets, the feature names are described by the names of referential species, and binary vectors are values of these features.

### 2.3. Method of Geptop

Geptop calculates gene essentiality through combining the features which computed by homology mapping with corresponding evolutionary distance between query species and its referential species. The evolutionary distance is calculated by composition vector (CV) [25]; it is employed as the weight of orthologous-essential gene. For a given genome, Geptop took multiple homology mapping to obtain the orthologous-essential genes of different referential species. Then, it computed the CV distances between the query genome and the other referential ones. The gene essentiality of the given organism is calculated by the following equation:

(1) $$\begin{array}{c} S_{i}=1-{\left(\;\sum_{j=1}^{N}E_{ij}\times D_{j}\;\right)}^{1/N},\;\left(j=1,2,3,\ldots ,N\right), \end{array}$$

where *S*_*i*_ represents the essentiality score of *i*th gene of query species, *E*_*ij*_ represents the essentiality of the optimal orthologous gene of *i*th query gene in *j*th referential species, *D*_*j*_ is the distance between the query species and the *j*th referential species, and *N* is the total number of referential species. Thus, Geptop method can decide whether a gene is essential or not according to the essentiality score.

### 2.4. Method Based on Sequence Feature and Machine Learning Algorithm

#### 2.4.1. Support Vector Machine

Support vector machine (SVM) [26], an efficient machine learning method, has been widely used in classification and pattern recognition. It adopts the principle of structural risk minimization and belongs to supervised learning method. SVM maps features into a high-dimensional feature space by kernel function. In the high-dimensional space, the samples with different attributions can be separated easily. In the present work, we adopted LibSVM [27] to perform SVM algorithm with RBF kernel function. It gave evaluation index for each feature that differed from the weight given by Geptop method.

#### 2.4.2. Feature Selection

In order to measure the contribution of each feature in a test process, we utilized *F*-score algorithm [28] to estimate the importance of them. *F*-score is a simple and quite effective arithmetic to discriminate two sets of real data. The larger the score is, the more significant contribution the feature makes. For training vectors *x*_*k*_(*k* = 1, 2, …, *p*) the *F*-score is defined as followings:

(2) $$\begin{array}{c} F\left(\;i\;\right)=\frac{{\left({\overset{-}{x_{i}}}^{\left(+\right)}-\overset{-}{x_{i}}\right)}^{2}+{\left({\overset{-}{x_{i}}}^{\left(-\right)}-\overset{-}{x_{i}}\right)}^{2}}{\left(1/\left(n_{+}-1\right)\right)\sum_{k=1}^{n_{+}}{\left(x_{k,i}^{\left(+\right)}-{\overset{-}{x}}_{i}^{\left(+\right)}\right)}^{2}+\left(1/\left(n_{-}-1\right)\right)\sum_{k=1}^{n_{-}}{\left(x_{k,i}^{\left(-\right)}-{\overset{-}{x}}_{i}^{\left(-\right)}\right)}^{2}}, \end{array}$$

where *n*_+_ and *n*_−_ are the number of positive and negative samples, respectively; $\overset{-}{x_{i}}$, ${\overset{-}{x_{i}}}^{\left(+\right)}$, and ${\overset{-}{x_{i}}}^{\left(-\right)}$ are the mean of the *i*th feature of the total, positive and negative samples, respectively; *x*_*k*,*i*_^(+)^ is the *i*th feature of the *k*th positive sample; *x*_*k*,*i*_^(−)^ is the *i*th feature of the *k*th negative sample.

#### 2.4.3. Classifier Design and Performance Evaluation

For a species under test, the homology mapping was implemented between the query species and other 24 organisms, and then 24 features were obtained to train the classifier. We used the classic machine learning method SVM to train the model and predict essential genes. Gaussian kernel function was selected to project the original features into a high-dimensional space. Gridding search method was adopted to search the best penalty parameter *C* and *γ*. Cross-validation and receiver operating characteristic (ROC) curve is the usual performance evaluation method for predictions [29]. Therefore, we used the area under ROC curve (AUC) of tenfold cross-validation to evaluate the performance of our classifier. For 10-fold cross-validation, the training data were randomly divided into 10 equal parts. Nine parts were used to train the classifiers and the remaining part was used for testing. This process was repeated until each part was taken as test set. For prediction in cross organisms, we chose the feature sets of the closest organism or them of the greatest contributed feature/organism to train the model and used the same characteristic variables of test organism to make prediction. The predictions were compared with the known gene essentialities which have been determined by experimental method.

## 3. Results

### 3.1. Evolutionary Distance and Orthology in Cross Species

If two organisms have closer evolutionary distance, they may share more orthologous genes or more common essential genes, relatively. We used an online web server CVTree [30] to establish phylogenetic tree for the selected 25 species. The phylum each organism belongs to and the distance relationships among them were illustrated in Supplementary Data Figure 1 in Supplementary Material available online at http://dx.doi.org/10.1155/2016/7639397. We discovered that only one organism belongs to* Actinobacteria*, other 24 organisms are from 4 different phyla, and each phylum has more than one organism.

**Figure 1:**
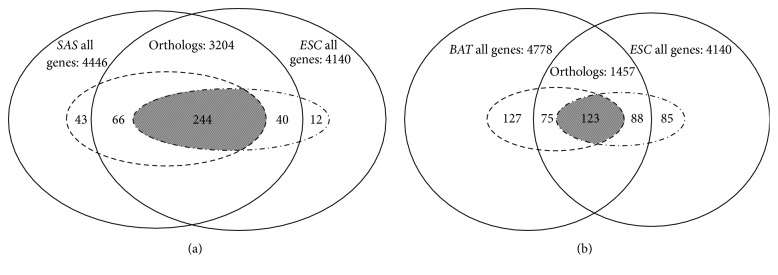
Comparison of the number of conserved genes and essential genes between two organisms. (a) We compared the difference between* SAS* and* ESC*, two relatively closely related organisms. They shared 3204 orthologous genes and 244 common essential genes. The broken circle represents 353* SAS* essential genes, and the dash dotted line circle represents 296* ESC* essential genes. For* ESC*, there are 310 orthologous-essential genes in* SAS*. (b) We compared the difference between* BAT* and* ESC*, two relatively distantly related organisms. They shared 1457 orthologous genes and 123 common essential genes. The broken circle represents 325* BAT* essential genes, and the dash dotted line circle represents 296* ESC* essential genes. For* ESC*, there are 198 orthologous-essential genes in* BAT*. Obviously, the closer species may have more orthologous sequence and more common essential genes with the target species than the distant one.

Essential genes are always inclined to be conserved because of their important functions, while conserved genes across species are not necessarily essential. We compared the number of conserved genes and essential genes between two organisms to illustrate the orthologous relationships across species (Figure 1). We analyzed these relationships in two groups. One pair contains relatively closely related organisms:* ESC* and* SAS* (Figure 1(a)), and the other pair contains relatively distantly related organisms:* ESC* and* BAT* (Figure 1(b)). The evolutionary distances of* SAS*-*ESC* and* BAT*-*ESC* are 0.3273 and 0.4976, respectively. There are 3204 orthologous genes between* ESC* and* SAS*, in which there are 244 common essential genes, accounting for around 77.39% and 5.89% in the total number of* ESC* genes, respectively. In the other group, 1457 genes are orthologous between* ESC* and* BAT*, in which there are 123 common essential genes, accounting for only 35.19% and 2.97% in the total number of* ESC* genes, respectively. These two results exemplified that the closer the evolutionary distance between species is, the more orthologous genes or common essential genes they would share. Meanwhile, an organism may share different essential genes with different organisms. Hence, we need to use multiple genomes to implement homology mapping, which we called multiple homology mapping.

### 3.2. Classifier Training towards 25 Genomes and 10-Fold Cross-Validations of the Classifier

**Figure 2:**
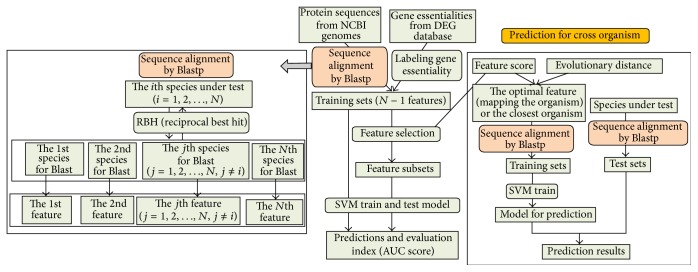
The flowchart for obtaining training sets by multiple homology mapping and training the model to predict essential genes. For a species under test, it was used for sequence alignment towards other 24 species, respectively, and each result was used as a training feature. The training sets obtained from multiple sequence alignment were used to train and test the prediction model by SVM. Meanwhile, we used the *F*-score to evaluate the discriminative capability of each feature. The optimal feature subsets were selected to train and test the model. Tenfold cross-validation was utilized to assess the performance of the classifier. For predicting essential genes in cross organisms, the feature sets of the closest organism or those of the organism/feature which has the biggest *F*-score for the target species were selected as the training sets to train model, and then this model was used to predict essential genes in target species.

**Figure 3:**
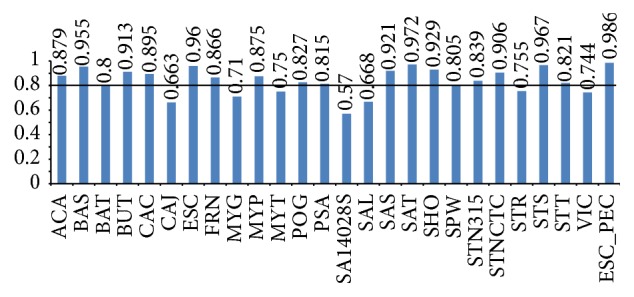
26 AUC scores of 10-fold cross-validation within 25 bacteria as well as* ESC_PEC*, respectively. The last AUC score belongs to* ESC* whose data are obtained from PEC database. More than 70% of the results exceed the AUC score of 0.80, and 9 organisms' results of prediction yielded AUC scores more than 0.90.

We gave the flowchart to display how this work was implemented in Figure 2. In this study, features we used were derived from protein sequences via homology mapping, which are easier to be acquired compared with other various biological features. Totally, 24 features were used as input variables for SVM classifier. The input data contained 24 features and the class labels for the species under test. Each genome was taken as the test species, and each of their AUC score of 10-fold cross-validations was acquired. All of their results of prediction yielded AUC scores between 0.5700 and 0.9716 (Figure 3) and accuracy scores between 0.7980 and 0.9805 (Supplementary Data Table 1). In addition, the AUC score of* ESC_PEC (E. coli)* whose data were obtained from PEC database is 0.9864 by this classifier. Previously, Deng et al. [12] got AUC score of 0.93 by 10-fold cross-validation for the same dataset through combining 4 machine learning methods and 13 features including codon bias index, Aromaticity, and Paralogy. Absolutely, our method achieved better performance than theirs. In addition, more than 70% of the bacteria exceed AUC score of 0.80, and merely 12% of all are less than 0.70. These results demonstrate that our classifiers have quite great performance.

### 3.3. Predictions after Feature Selection

In the above work, all features obtained by homology mapping were utilized to train the classifiers, but not every feature has high predictive power for identification of essential genes. Therefore, ranking the features in order and filtering out the useless features played an important role in prediction [29]. We used the method of feature selection to choose appropriate feature subsets to reappraise the performance of classifiers. Based on *F*-score algorithm [28], we got a vector composed of 24 feature scores in descending order for an organism under test. Another feature selection method named DX score [31] was also applied to measure feature score, and the same order of features were acquired. It indicated that the features' order we obtained was reliable. Feature subsets were constructed through appending a feature one by one in accordance with the descending order, and each feature subset was utilized to train and test the model. Finally, the optimal feature subset and its predictions would be chosen on the basis of AUC score. After selecting optimal feature subsets, most of predictions have corresponding improvement. The AUC scores of 8 species were improved by more than 1%, and the average AUC score was improved around 1% among 25 species comparison with the results of using all features. In addition, contribution score of each feature was obtained by *F*-score. For each organism under test, we analyzed the correlation between feature scores and evolutionary distances through Pearson correlation analysis (Table 2). We discovered that 22 species among all presented negative correlations, in which 15 results presented significant negative correlations. That is to say the importance of features evaluated by machine learning method is consistent with the evolutionary distance between them in general.

### 3.4. Predicting Essential Genes across Organisms

**Figure 4:**
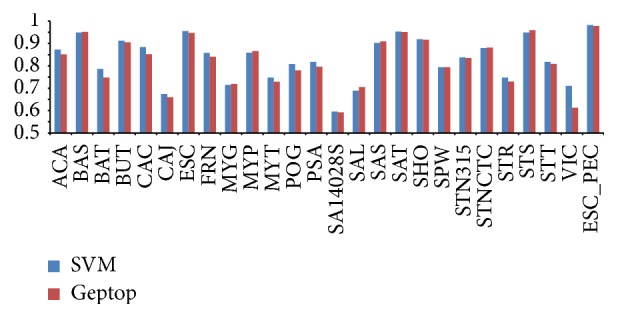
Comparison AUC scores of interspecies prediction for 25 bacteria between SVM and Geptop. The last AUC score belongs to* ESC* whose data are obtained from PEC database. The vertical axis, in the range from 0.5 to 1, represents AUC scores. More than 65% of the results exceed the AUC score of 0.80, and 8 organisms' results of prediction yielded AUC scores more than 0.90. For 26 genomes including* ESC_PEC*, 18 of all are better than Geptop.

It is necessary to use the suitable model to predict essential genes across distantly related bacteria. The relatively closely related organisms may have similar patterns in developing model for predicting gene essentiality when using orthologs to some extent. For a species with unknown gene essentiality, using the features of closest organism as training set to train model is available. However, the closest species may not be the most important contributor in machine learning methods. Besides, if the data quality of this species is poor, it could cause bad effects for the classifier and the classifier could not give the best predictions. Therefore, we chose the characteristic set of closest species and the characteristic set of the greatest contributor to train the model, respectively. The better outputs of these diverse training set were chosen as the results of prediction. For example,* Salmonella enterica *serovar Typhimurium LT2* (SAL)* is the closest related bacterium for* ESC*, but the classifier built by its features made a low AUC score of 0.7894. This may be related to the low data quality of* SAL* itself (Supplementary Data Table 2). Nevertheless, we chose the features of* Salmonella enterica* serovar Typhi SL1344* (SAS)* as training set to train model for predicting essential genes of* ESC*, because feature SAS has maximum *F*-score among all features of* ESC*. AUC score of 0.9552 and precision (or PPV) of 0.7330 were acquired, and the classifier identified 258 true essential genes from its 352 positive outputs. These two values are greater than the AUC score of 0.9470 and precision (or PPV) of 0.6574 through Geptop method, which identified 236 true essential genes from its 359 positive outputs. We applied this method to implement interspecies prediction among other 24 organisms. As a result, AUC scores between 0.5957 and 0.9552 were obtained for 25 bacteria except* ESC_PEC* (Figure 4, Supplementary Data Table 3). For 26 genomes including* ESC_PEC*, 18 results of prediction are better than Geptop. And the average AUC was improved by 1.14% compared with Geptop.

**Figure 5:**
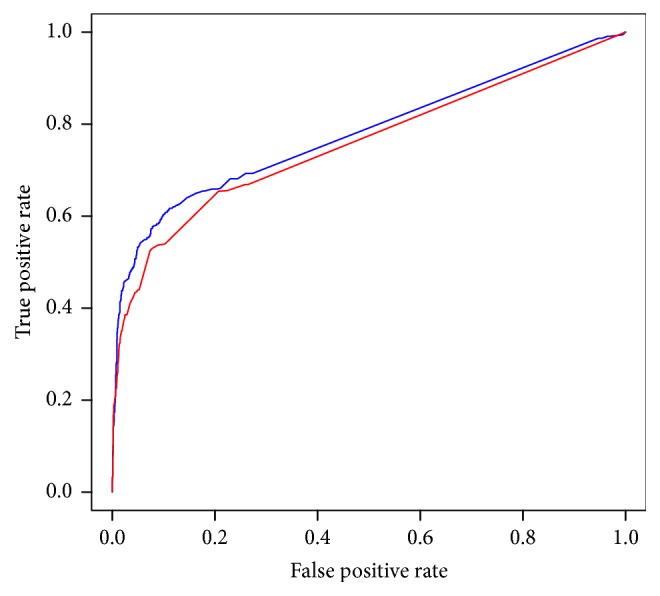
Comparison the ROC curve of Geptop and SVM for* SYE*. The blue curve represents the results obtained through SVM, and the area under it is 0.7855. The red curve represents the results obtained through Geptop, and the area under it is 0.7578.

A newly determined essential gene set of* Synechococcus elongatus* PCC 7942* (SYE)* by experimental technology are acquirable for independent testing [32]. Totally, 674 genes are annotated as essential in its genome. This bacterium belongs to* Cyanophyta*, a quite different phylum compared with the bacteria already used in this study. We collected this dataset to further evaluate the performance of our classifier. The evolutionary distances between* SYE* and the existing organisms were calculated by composition vector (CV) method. The nearest species was determined as* Caulobacter crescentus* NA1000* (CAC)*. We chose the features of* CAC* to train a classifier and obtained values of the same features of* SYE* as test set through homology mapping between it and other 24 species except* CAC*. Through SVM, we achieved AUC score of 0.7855 (Figure 5) and precision (or PPV) of 0.8105. In order to assess performance of machine learning method, Geptop method was implemented to predict essential genes on* SYE*, and then it obtained AUC score of 0.7578 and precision (or PPV) of 0.8484. Although the precision of Geptop is higher than that of SVM, SVM method identified 325 true essential genes from its 401 positive outputs, which are more than 263 true essential genes identified by Geptop.

## 4. Discussion

We make predictions based on gene essentialities which have been determined by experimental technology. There is no doubt that the results of prediction would be influenced by quality of experimental data. For example, if insertion was avoided accidentally, transposon mutagenesis technique would be likely to mislabel short genes [33]. Therefore, the reason why those three species* (CAJ, SAL*, and* SA14028S)* have AUC lower than 0.70 may be that a mass of essential genes were mislabeled when they were identified by experimental techniques. This has been discussed in Geptop. Additionally, according to the fact that few contributions* SA14028S* provided for almost all organisms and few contributions these features provided for it, we can further presume that the data of this species have low quality.

Geptop gave weights for features only based on evolutionary distance, which would ignore the data quality. Our machine learning method measures the features from an integrated view. In order to investigate the reason why our method performs better than Geptop, we ranked the features based on theirs abilities of differentiate between positive and negative sample sets, which were measured by *F*-score in our method, and we also ranked them based on weights which were measured by evolutionary distance in Geptop. The rank changes of features from Geptop to SVM were calculated. We analyzed the relationships between AUC scores of 10-fold cross-validation and rank changes using Pearson correlation method (Table 3). We find that 23 among 25 results present significant positive correlations. It indicates that rank changes of features from Geptop to SVM are consistent with AUC values. Taking* ACA* as example (Supplementary Data Table 4), the most important feature in Geptop is ranked as 11th in SVM. Specially, the 6th important feature in Geptop is ranked as 24th in SVM. In addition, this feature has no contribution for predicting. In truth, the 24th feature for* ACA* in SVM is* SA14028S*, which has been discussed that it has low data quality in the above paragraph. Furthermore, the important features in Geptop with relatively high AUC like* ESC* and* SAS* have no or few rank changes. In other words, our method can take the predictive power of every feature into full account. Simultaneously, as a machine learning method, SVM has its inherent advantages. For instance, the final decision function is dominantly determined by a few support vectors, which not only can help us acquire key samples, but also has good robustness. Thus, our method can achieve better predictions than Geptop; the latter makes prediction completely depending on biological knowledge.

## 5. Conclusion

Our classifier is designed based on RBH method, which can reflect substantive characteristics of orthologous sequence. Although the homology mapping method may ignore the species-specific essential genes, it still can identify reasonable number of essential genes. Superiority of multiple homology mapping has been presented in Geptop, which acquired satisfactory results in predicting essential genes. Besides, these features could be extracted for almost all sequenced bacterial genomes. We utilized this method incorporating with SVM to train classifier and predict essential genes, and then the classifier achieved better performance than Geptop. It probably gives the credit to the fact that our method can measure predictive ability of each feature through machine learning method, which differs from Geptop that only considers evolutionary distance; thus our method can train the better model. Geptop has been designed as a web server; our method also has potential to be developed as a tool to provide service for users.

In conclusion, through multiple homology mapping and machine learning method, we provide a significant alternative method to predict essential genes. The results of prediction yield higher AUC scores than those integrated approaches as well as Geptop method. Simultaneously, this work reveals that machine learning method may perform better than the method using empirical formula; the latter was developed completely based on biological knowledge. With more reliable and available experimental essential gene sets, the performance of our method will be improved to an even better level.

## Supplementary Material

The Supplementary material lists detailed information of main results of this work in PDF document format, including accuracy scores of 10-fold cross-validation, the evolutionary distance of *ESC* toward other 24 species and F-scores of 24 features of *ESC*, AUC scores of interspecies prediction obtained through SVM and Geptop method respectively, the feature ranks of organism *ACA* acquired through SVM and Geptop method respectively, as well as phylogenetic tree for the selected 25 organisms.

## Figures and Tables

**Table 1:** Bacteria used in this work.

Species	Abbreviation	Number of essential genes	Number of total genes
*Acinetobacter *ADP1	*ACA*	499	3307
*Bacillus subtilis *168	*BAS*	271	4175
*Bacteroides thetaiotaomicron *VPI 5482	*BAT*	325	4778
*Burkholderia thailandensis *E264	*BUT*	406	5632
*Caulobacter crescentus *NA1000	*CAC*	480	3885
*Campylobacter jejuni *NCTC 11168 ATCC 700819	*CAJ*	222	1572
*Escherichia coli *K-12 MG1655	*ESC*	296	4140
*Escherichia coli *K-12 in PEC database	*ESC_PEC*	287	4146
*Francisella novicida *U112	*FRN*	390	1719
*Mycobacterium tuberculosis *H37Rv	*MYT*	611	3906
*Mycoplasma genitalium *G37	*MYG*	378	475
*Mycoplasma pulmonis *UAB CTIP	*MYP*	310	782
*Porphyromonas gingivalis *ATCC 33277	*POG*	463	2089
*Pseudomonas aeruginosa *UCBPP PA14	*PSA*	335	5892
Salmonella enterica serovar Typhimurium 14028S	*SA14028S*	105	5315
Salmonella enterica serovar Typhimurium LT2	*SAL*	230	4451
Salmonella enterica serovar Typhimurium SL1344	*SAS*	353	4446
Salmonella enterica serovar Typhi Ty2	*SAT*	358	4352
*Shewanella oneidensis *MR 1	*SHO*	402	4065
*Sphingomonas wittichii *RW1	*SPW*	535	4850
*Staphylococcus aureus *N315	*STN315*	302	2582
*Staphylococcus aureus *NCTC 8325	*STNCTC*	346	2767
*Streptococcus pneumonia *TIGR4	*STT*	111	2105
*Streptococcus pneumonia *R6	*STR*	127	1814
*Streptococcus sanguinis *SK36	*STS*	218	2270
*Vibrio cholerae *O1 biovar El Tor N16961	*VIC*	591	3503

The number of essential genes and total genes are counted after filtering unmatched data.

**Table 2:** Correlations between evolutionary distances and feature scores for each target organism.

Organisms	Correlations	*P* value
*ACA* ^*∗*^	−0.45204	0.0266
*BAS* ^*∗∗*^	−0.37043	0.0075
*BAT* ^*∗*^	−0.50001	0.0128
*BUT* ^*∗*^	−0.41124	0.0459
*CAC* ^*∗*^	−0.41482	0.0439
*CAJ*	−0.23786	0.2631
*ESC* ^*∗*^	−0.50218	0.0120
*ESC_PEC* ^*∗∗*^	−0.52353	0.0087
*FRN*	−0.39292	0.0575
*MYT*	−0.35883	0.0851
*MYG* ^*∗*^	−0.49728	0.0134
*MYP* ^*∗∗*^	−0.54766	0.0056
*POG* ^*∗*^	−0.46123	0.0233
*PSA* ^*∗∗*^	−0.60836	0.0016
*SA14028S* ^*∗∗*^	−0.60533	0.0017
*SAL*	−0.28669	0.1744
*SAS*	−0.24910	0.2405
*SAT*	−0.31248	0.1371
*SHO* ^*∗*^	−0.50456	0.0119
*SPW* ^*∗∗*^	−0.65577	0.0005
*STN315*	−0.11162	0.6036
*STNCTC*	0.03883	0.8570
*STR*	0.11619	0.5887
*STS*	0.24868	0.2413
*STT*	0.19591	0.3589
*VIC* ^*∗*^	−0.50718	0.0114

*∗* represents that the correlation is significant at the 0.05 level; *∗∗* represents that the correlation is significant at the 0.01 level.

**Table 3:** Correlations between rank changes and AUC scores of 10-fold cross-validation.

Organisms	Correlations	*P* value
*ACA* ^*∗∗*^	0.66774	0.0005
*BAS* ^*∗*^	0.50475	0.0140
*BAT* ^*∗∗*^	0.61735	0.0017
*BUT* ^*∗∗*^	0.64683	0.0009
*CAC* ^*∗∗*^	0.64730	0.0008
*CAJ* ^*∗∗*^	0.52945	0.0094
*ESC* ^*∗∗*^	0.69090	0.0003
*FRN* ^*∗∗*^	0.70211	0.0002
*MYT* ^*∗∗*^	0.58115	0.0036
*MYG* ^*∗∗*^	0.58017	0.0037
*MYP* ^*∗∗*^	0.67868	0.0004
*POG* ^*∗∗*^	0.58558	0.0033
*PSA* ^*∗∗*^	0.62930	0.0013
*SA14028S*	0.36461	0.0872
*SAL* ^*∗∗*^	0.66214	0.0006
*SAS* ^*∗∗*^	0.69220	0.0003
*SAT* ^*∗∗*^	0.70091	0.0002
*SHO* ^*∗∗*^	0.73831	5.77*E* − 05
*SPW* ^*∗∗*^	0.66206	0.0006
*STAN315* ^*∗*^	0.50613	0.0137
*STANCTC* ^*∗∗*^	0.54941	0.0066
*STR*	0.37110	0.0813
*STS* ^*∗∗*^	0.58267	0.0035
*STT* ^*∗∗*^	0.70091	0.0002
*VIC* ^*∗∗*^	0.65402	0.0007

*∗* represents that the correlation is significant at the 0.05 level; *∗∗* represents that the correlation is significant at the 0.01 level.
