# Supplementary material for: An Approach for Predicting Essential Genes Using Multiple Homology Mapping and Machine Learning Algorithms
Source: Biomed Res Int. 2016 Aug 30;2016:7639397. doi: 10.1155/2016/7639397 (PMC5021884; doi:10.1155/2016/7639397)
Supplement: Supplementary file 1 — The Supplementary material lists detailed information of main results of this work in PDF document format, including accuracy scores of 10-fold cross-validation, the evolutionary distance of ESC toward other 24 species and F-scores of 24 features of ESC, AUC scores of interspecies prediction obtained through SVM and Geptop method respectively, the feature ranks of organism ACA acquired through SVM and Geptop method respectively, as well as phylogenetic tree for the selected 25 organisms. [file 7639397.f1.docx]

**Table S1.** Accuracy (ACC) scores of 10-fold cross-validation within 26 genomes including *ESC_PEC*, respectively

| Species | Accuracy | Species | Accuracy |
| --- | --- | --- | --- |
| *ACA* | 0.9256 | *PSA* | 0.9537 |
| *BAS* | 0.9689 | *SA14028S* | 0.9802 |
| *BAT* | 0.9481 | *SAL* | 0.9486 |
| *BUT* | 0.9602 | *SAS* | 0.9746 |
| *CAC* | 0.9364 | *SAT* | 0.9805 |
| *CAJ* | 0.8594 | *SHO* | 0.9582 |
| *ESC* | 0.9756 | *SPW* | 0.9173 |
| *ESC_PEC* | 0.9846 | *STN315* | 0.9237 |
| *FRN* | 0.8819 | *STNCTC* | 0.9422 |
| *MYG* | 0.7980 | *STR* | 0.9355 |
| *MYP* | 0.8363 | *STS* | 0.9568 |
| *MYT* | 0.8784 | *STT* | 0.9539 |
| *POG* | 0.8760 | *VIC* | 0.8767 |

**Table S2.** The evolutionary distances of *ESC* toward other 24 species, and F-scores of 24 training features, respectively

| Organisms | Distances | F-scores | Organisms | Distances | F-scores |
| --- | --- | --- | --- | --- | --- |
| *ACA* | 0.490356 | 0.436212 | *SA14028S* | 0.328889 | 0.000473 |
| *BAS* | 0.496555 | 0.438963 | *SAL* | 0.325947 | 0.107973 |
| *BAT* | 0.497623 | 0.296408 | *SAS* | 0.327302 | 1.662053 |
| *BUT* | 0.492183 | 0.444045 | *SAT* | 0.328520 | 1.580226 |
| *CAC* | 0.495776 | 0.445921 | *SHO* | 0.483109 | 0.731280 |
| *CAJ* | 0.497385 | 0.098472 | *SPW* | 0.496202 | 0.177434 |
| *FRN* | 0.494585 | 0.511715 | *STAN315* | 0.496979 | 0.199663 |
| *MYG* | 0.499252 | 0.154263 | *STANCTC* | 0.496908 | 0.354130 |
| *MYP* | 0.499072 | 0.188904 | *STR* | 0.496926 | 0.051073 |
| *MYT* | 0.497795 | 0.173656 | *STS* | 0.497187 | 0.353390 |
| *POG* | 0.497714 | 0.303464 | *STT* | 0.497592 | 0.066151 |
| *PSA* | 0.488101 | 0.290296 | *VIC* | 0.476311 | 0.430859 |

**Table S3.** Comparison AUC scores of interspecies prediction between SVM and Geptop.

| Organisms | AUC of SVM | AUC of Geptop | Organisms | AUC of SVM | AUC of Geptop |
| --- | --- | --- | --- | --- | --- |
| *ACA* | 0.873 | 0.851 | *PSA* | 0.818 | 0.796 |
| *BAS* | 0.949 | 0.952 | *SA14028S* | 0.596 | 0.592 |
| *BAT* | 0.786 | 0.748 | *SAL* | 0.689 | 0.705 |
| *BUT* | 0.912 | 0.905 | *SAS* | 0.903 | 0.909 |
| *CAC* | 0.884 | 0.852 | *SAT* | 0.953 | 0.951 |
| *CAJ* | 0.674 | 0.660 | *SHO* | 0.919 | 0.917 |
| *ESC* | 0.955 | 0.947 | *SPW* | 0.794 | 0.794 |
| *ESC_PEC* | 0.982 | 0.978 | *STN315* | 0.838 | 0.835 |
| *FRN* | 0.858 | 0.841 | *STNCTC* | 0.879 | 0.882 |
| *MYT* | 0.748 | 0.729 | *STR* | 0.748 | 0.730 |
| *MYG* | 0.715 | 0.719 | *STS* | 0.949 | 0.959 |
| *MYP* | 0.859 | 0.866 | *STT* | 0.818 | 0.809 |
| *POG* | 0.808 | 0.780 | *VIC* | 0.710 | 0.613 |

**Table S4.** The ranks of two types of weights for *ACA*

| Features | Rank^G^ | Evolutionary distance | Rank^S^ | coefficient of SVM |
| --- | --- | --- | --- | --- |
| *BAS* | 13 | 0.496739 | 9 | 0.294521 |
| *BAT* | 22 | 0.498103 | 16 | 0.158641 |
| *BUT* | 9 | 0.492145 | 4 | 0.433081 |
| *CAC* | 12 | 0.495855 | 6 | 0.395008 |
| *CAJ* | 16 | 0.497303 | 21 | 0.063133 |
| *ESC* | 2 | 0.490356 | 5 | 0.401255 |
| *FRN* | 10 | 0.494423 | 7 | 0.383845 |
| *MYG* | 23 | 0.499379 | 19 | 0.103328 |
| *MYP* | 24 | 0.499406 | 18 | 0.135161 |
| *MYT* | 19 | 0.497532 | 10 | 0.274782 |
| *POG* | 21 | 0.497932 | 15 | 0.192427 |
| *PSA* | 1 | 0.487977 | 11 | 0.268717 |
| *SA14028S* | 6 | 0.491137 | 24 | 0 |
| *SAL* | 5 | 0.491001 | 20 | 0.086136 |
| *SAS* | 4 | 0.490949 | 2 | 0.458404 |
| *SAT* | 3 | 0.490816 | 1 | 0.477181 |
| *SHO* | 8 | 0.491818 | 3 | 0.442998 |
| *SPW* | 11 | 0.495726 | 8 | 0.380825 |
| *STN315* | 14 | 0.497207 | 17 | 0.142647 |
| *STNCTC* | 15 | 0.497236 | 14 | 0.23916 |
| *STR* | 18 | 0.497341 | 22 | 0.056178 |
| *STS* | 17 | 0.497321 | 13 | 0.242034 |
| *STT* | 20 | 0.497904 | 23 | 0.056178 |
| *VIC* | 7 | 0.491719 | 12 | 0.266336 |

Rank^G^ represents the orders of features in Geptop; Rank^S^ represents the orders of features in SVM.


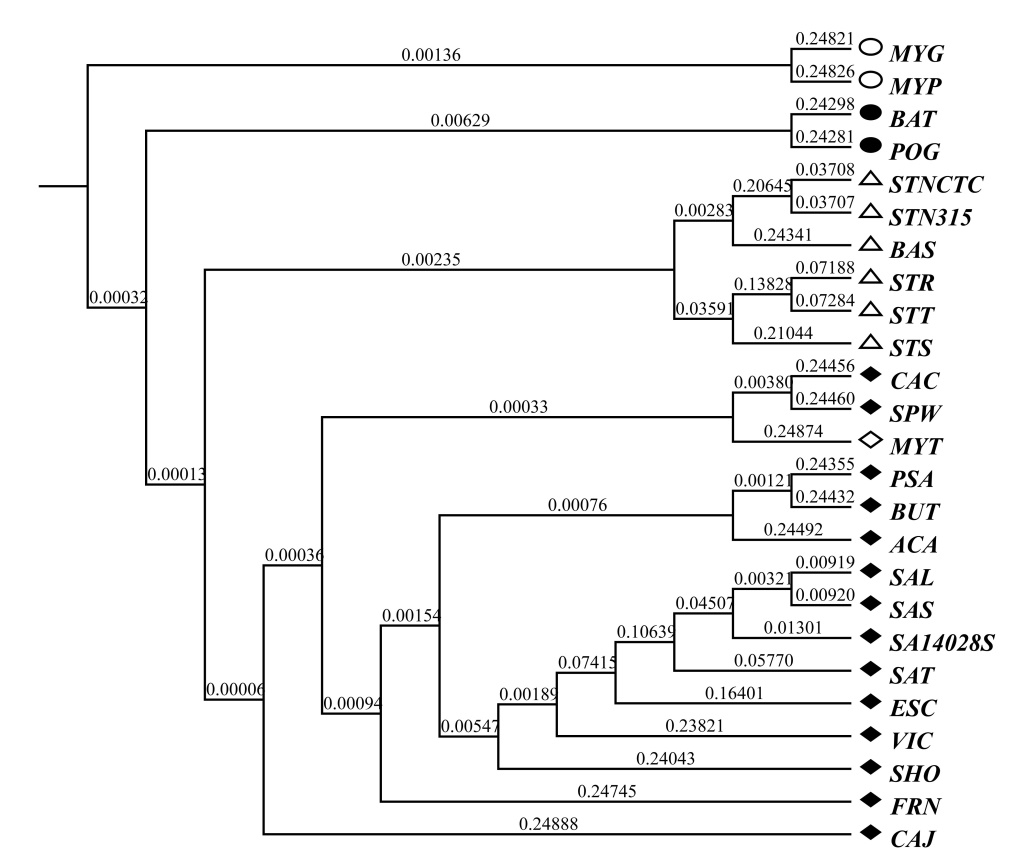


**Figure S1. Phylogenetic tree for the selected 25 species.** White circle represents *Tenericutes*; black circle represents *Bacteroidetes*; white triangle represents *Firmicutes*; white diamond represents *Actinobacteria*; black diamond represents *Proteobacteria*.
